# The Study of Nanosized Silicate-Substituted Hydroxyapatites Co-Doped with Sr^2+^ and Zn^2+^ Ions Related to Their Influence on Biological Activities

**DOI:** 10.3390/cimb44120425

**Published:** 2022-12-09

**Authors:** Justyna Rewak-Soroczynska, Nicole Nowak, Sara Targonska, Agata Piecuch, Rafal J. Wiglusz

**Affiliations:** 1 Institute of Low Temperature and Structure Research, PAS, ul. Okolna 2, PL-50-422 Wroclaw, Poland; 2 Department of Animal Biostructure and Physiology, Wroclaw University of Environmental and Life Sciences, Norwida 25, PL-50-375 Wroclaw, Poland; 3 Department of Mycology and Genetics, University of Wroclaw, Przybyszewskiego 63, PL-51-148 Wroclaw, Poland

**Keywords:** nanosilicate-substituted hydroxyapatite, Sr^2+^ and Zn^2+^ doping, antibacterial activity, antibiofilm activity, cytotoxicity

## Abstract

Nanosized silicate-substituted hydroxyapatites, characterized by the general formula Ca_9.8−x−n_Sr_n_Zn_x_(PO_4_)_6−y_(SiO_4_)_y_(OH)_2_ (where: n = 0.2 [mol%]; x = 0.5–3.5 [mol%]; y = 4–5 [mol%]), co-doped with Zn^2+^ and Sr^2+^ ions, were synthesized with the help of a microwave-assisted hydrothermal technique. The structural properties were determined using XRD (X-ray powder diffraction) and Fourier-transformed infrared spectroscopy (FT-IR). The morphology, size and shape of biomaterials were detected using scanning electron microscopy techniques (SEM). The reference strains of *Klebsiella pneumoniae*, *Escherichia coli* and *Pseudomonas aeruginosa* were used to assess bacterial survivability and the impact on biofilm formation in the presence of nanosilicate-substituted strontium-hydroxyapatites. Safety evaluation was also performed using the standard cytotoxicity test (MTT) and hemolysis assay. Moreover, the mutagenic potential of the materials was assessed (Ames test). The obtained results suggest the dose-dependent antibacterial activity of nanomaterials, especially observed for samples doped with 3.5 mol% Zn^2+^ ions. Moreover, the modification with five SiO_4_ groups enhanced the antibacterial effect; however, a rise in the toxicity was observed as well. No harmful activity was detected in the hemolysis assay as well as in the mutagenic assay (Ames test).

## 1. Introduction

In recent years, the overuse of antibiotics has caused the development of microbial resistance to numerous commonly applied formulations. Apart from drugs, resistance to some antimicrobial agents (disinfectants) has been also reported over the years [1,2,3]. Bacteria and fungi have developed many virulence mechanisms that allow them to not only actively invade the host body but also to remain persistent and less prone to harmful chemical and physical conditions. Among such factors is biofilm, which is described as microbial community surrounded by the extracellular matrix which protects the cells [4]. Metal ions seemed to be the most promising solution to combat drug-resistant strains, but some of them, especially at higher concentrations, are cytotoxic (in vitro) towards eukaryotic cells. Moreover, applying sublethal doses may also lead to the development of resistance mechanisms among pathogens. Silver ions are known for their strong antimicrobial activity. However, the bacterial tolerance to formulations containing Ag^+^ ions has been already reported [5,6,7,8]. The reduction of ion concentration and, subsequently, its cytotoxicity, without decreasing antibacterial effects, may be obtained by combining various ions [9,10,11,12,13,14,15].

One possible way of using more than one metal ion simultaneously is by co-doping a hydroxyapatite matrix. Hydroxyapatite (HAp) naturally occurs in the human body as a component of bones and teeth. However, it can also be found in other organisms, e.g., in anthozoa [16]. Its strong biocompatibility with osteoblasts and osteoclasts in the cell culture is related to its high porosity and structural similarity to natural bone. A beneficial influence on bone growth after HAp scaffold implantation has also been reported [5,17]. Combining apatites with antimicrobial ions results in obtaining a non-toxic, biocompatible porous material with the ability to prevent microbial growth. Apart from silver, which is often described as genotoxic and cytotoxic, such metals as gold, zinc, magnesium, iron or copper are reported to possess antimicrobial activity, especially against Gram-negative species, generally considered as more resistant to common antimicrobials [5,17,18,19]. On the other hand, it has been proved that nanoparticles are less active against Gram-positive bacteria. This phenomenon is attributed to a thicker cell wall in Gram-positive strains, which acts as a barrier for nanoparticles penetration. Moreover, a negative charge on the surface of Gram-negative bacteria, conferred by the lipopolysaccharide, leads to the higher affinity for cations [20]. The possible mechanisms of metal ion activity is widely studied and their antibacterial effects are attributed mainly to the ability of protein (including enzymes) impairment, reactive oxygen species production (leading to oxidative stress), cellular membrane disruption and genetic material damages [21]. 

As already mentioned, zinc ions exhibit antimicrobial activity that affects both Gram-positive and Gram-negative species, including *Staphylococcus epidermidis*, *S. aureus*, *Streptococcus mutans*, *Pseudomonas aeruginosa* and *Escherichia coli*. Moreover, Zn^2+^ ions were reported to reduce *Candida albicans* biofilm [5,22,23]. Zn^2+^-doped HAp also promotes osteoblasts proliferation and viability [5,17,24,25]. Recent studies on zinc-based materials gained more attention due to its application as biocompatible bioglasses, alloys, ceramics etc. Moreover, Zn^2+^ ions are important factors regulating cell metabolism and the expression of genes involved in bone differentiation and mineralization processes. Therefore, when Zn^2+^ ions are maintained in physiological and relatively low concentrations, cells function properly; however, higher concentrations can lead to cell death and increased cytotoxicity [26,27]. Strontium is another example of a metal reducing bacterial growth. However, its antimicrobial activity is not as high as that of the aforementioned metal ions. On the other hand, its good biocompatibility with osteoblasts and its ability to alter the physicochemical properties of compounds (such as solubility enhancement) indicates that it could be a good co-dopant [5,17,28,29,30,31]. The research on co-doped HAp materials is still rather scarce, compared to those with a single dopant, thus the cross-reactions between different ions are not yet entirely understood [14,32]. In addition to cationic substitution, HAp can also undergo anionic substitution. Partial replacement of the phosphate groups with silicate alters the physicochemical properties of the apatite but its impact on antimicrobial activity is not fully characterized. There are some reports suggesting its positive impact on mesenchymal stem cells derived from human adipose tissue when combined with Ag^+^-HAp. Ag/Si-Hap also causes a significant reduction in bacterial adhesion [33]. Moreover, silica nanoparticles are a well-known material in biological and medical applications, therefore they are widely used in cosmetics and the food industry, but also in, e.g., car engineering or as a paint component [34]. Surprisingly, the prolonged exposure of silica nanoparticles on the skin, oral tract and even the respiratory system is confirmed to be safe. Silica nanoparticles are mainly absorbed in the lungs and intestinal tract, and through that can be transported to and accumulated in the internal organs, yet no damage was found in any tissue of the brain, heart, lungs, liver, kidney, large intestine, spleen, heart, stomach and small intestine, when tested in vivo [35,36]. 

Properties such as biocompatibility and the safety of the substances with the ability to promote tissue regeneration are desirable in implantology. Moreover, the ability to reduce bacterial growth and adhesion is an additional advantage of such materials, since the surgical procedures carry a risk of an infection [37]. 

This work provides complex characterization of newly synthesized nanosilicate-substituted hydroxyapatites co-doped with Sr^2+^ and Zn^2+^ ions, including the evaluation of their antimicrobial activity against Gram-negative bacteria and cytotoxicity level. It should be highlighted that the combination of co-doping with Sr^2+^ and Zn^2+^ ions of the hydroxyapatite structure enriched by silicate groups is presented for the first time. 

## 2. Materials and Methods

### 2.1. Synthesis of the Nanosilicate-Substituted Strontium-Hydroxyapatite Powders

The nanosilicate-substituted hydroxyapatite doped with Sr^2+^ ions and co-doped with Zn^2+^ ions was synthesized using the hydrothermal wet technique. Ca(NO_3_)_2_∙4H_2_O (99.0–103.0% Alfa Aesar), (NH_4_)_2_HPO_4_ (>99.0% Acros Organics), tetraethyl orthosilicate TEOS (>99% Alfa Aesar), Sr(NO_3_)_2_ (99.0% min Alfa Aesar) and Zn(NO_3_)_2_∙6H_2_O (pure, Chempur, Poland) were used as substrates. The stoichiometric amounts of all starting substrates were dissolved in deionization water, then placed into a Teflon vessel and mixed. The pH was adjusted with ammonia (NH_3_∙H_2_O 25% Avantor, Poland) to obtain a pH = 10. The hydrothermal process was carried out in the microwave reactor (ERTEC MV 02-02) for 90 min at elevated temperature (250 °C) and under autogenous pressure (40–50 bar). The obtained nanocrystalline powders were centrifuged, rinsed several times with deionized water and dried. Afterwards, the materials were heat-treated in the range of 600–800 °C for 3 h to reject the amorphous phase and receive well-crystalized products with the chemical formula of Ca9.8_−_xnSrnZnx(PO_4_)6_−_y(SiO_4_)y(OH)_2_ (where: n = 0.2 [mol%]; x = 0.5–3.5 [mol%]; y = 4–5 [mol%]). The concentration of Sr^2+^ and Zn^2+^ was set in a ratio to entire calcium ions molar content in the following routine.

### 2.2. Characterization

X-ray diffraction was used to determine the crystalline structures of the obtained materials. X-ray diffraction patterns were carried out usinga PANalytical X’Pert Pro X-ray diffractometer equipped with the Ni-filtered Cu Kα1 radiation (Kα1 = 1.54060 Å, U = 40 kV, I = 30 mA) in the 2θ range of 10–60°. The EDS spectra were recorded to confirm the chemical formula. The EDS spectra and SEM images were measured usinga FEI Nova NanoSEM 230 scanning electron microscopy equipped with the EDS spectrometer (EDAX GenesisXM4) and operating at an acceleration voltage in the range of 3.0–15.0 kV and spot 2.5–3.0 were observed. The infrared spectra were measured using a Thermo Scientific Nicolet iS50 FT-IR spectrometer equipped with the Automated Beamsplitter exchange system (iS50 ABX containing DLaTGS KBr detector), built-in all-reflective diamond ATR module (iS50 ATR), Thermo Scientific Polaris™ and a HeNe laser was used as an IR radiation source. The FT-IR spectra were measured in KBr (FT-IR grade, ≥99% Sigma-Aldrich, St. Louis, MO, USA) pellets at room temperature in the range of 4000–400 cm^−1^ with a spectral resolution of 2 cm^−1^.

### 2.3. Antimicrobial Activity

Antimicrobial activity was determined using three reference Gram-negative strains: *Klebsiella pneumoniae* subsp. *pneumoniae* ATCC 700603, *Pseudomonas aeruginosa* ATCC 27853 and *Escherichia coli* ATCC 35218 (Department of Pathogen Biology and Immunology, University of Wroclaw). Bacterial cultures were incubated on nutrient agar plates for 24 h and colonies were transferred to saline (0.9% NaCl) to obtain a final bacterial concentration of 5 × 10^5^ CFU/mL. Bacterial suspensions were incubated for 18 h at 37 °C with shaking (120 rpm) in the presence of 10, 25, 50 and 100 µg/mL of the tested silicate-substituted strontium-hydroxyapatite (colloidal solutions prepared in the saline). Then, bacterial solutions were diluted and spread on the Mueller Hinton Agar for colony forming units (CFU/mL) evaluation after 18 h incubation at 37 °C. The statistical analysis of the results was performed using one-way-ANOVA and the Levene test, followed by the Tukey test in the OriginPro 2019b (OriginLab Corporation, Northampton, MA, USA) software (*p* < 0.05). 

### 2.4. Antibiofilm Activity

Antibiofilm activity was evaluated for materials with the highest Zn^2+^ dopants (3.5 mol%Zn^2+^/Sr^2+^:Si_4_-HAp and 3.5 mol%Zn^2+^/Sr^2+^:Si_5_-HAp), andthe sample without Zn^2+^ ion additive was used as a control. Powders were pressed to obtain pellets (∅ = 10 mm, mass = 150 mg) which (after UV sterilization) were incubated at 37 °C overnight with 1 mL of *Klebsiella pneumoniae* ATCC 700603 suspension in nutrient broth (optical density of 0.1) with shaking (120 rpm). The samples were gently rinsed with saline to remove non-adhered bacteria and dyed for 20 min with fluorescent dyes: SYTO 9 (λ_exc_ = 488 nm) and propidium iodide (λ_exc_ = 543 nm) (both at the concentration of 1 µL/mL) (LIVE/DEAD BacLight Bacterial Viability Kit, Invitrogen, Thermo Fisher Scientific, Waltham, MA, USA), rinsed and visualized in the confocal microscope (OlympusIX83 Fluoview FV 1200, magnification 20×).

### 2.5. Cell Cultures

The TC28A2 human chondrocyte cell line and 7F2 mouse osteoblast cell line were maintained in high glucose Dulbecco’s Modified Eagle Medium (DMEM) with L-glutamine (Biowest, Nuaillé, France) and supplemented with 10% Fetal Bovine Serum (FBS) South America Heat Inactivated (Biowest), 200 U/mL penicillin and 200 µg/mL streptomycin. TC28A2 and 7F2 cell cultures were incubated in standard conditions at 37 °C in humidified atmosphere of 5% CO_2_ and 95% air. Cell cultures were passaged three times, before experiments were conducted.

#### 2.5.1. Nanoparticles Stock Preparation

Stock dispersions of the tested silicate-substituted strontium-hydroxyapatite nanoparticles doped with Zn^2+^ ions and silica groups were prepared with a suspension of used compounds in distilled water. Then, each stock was bath-sonicated for 1 h at room temperature. Freshly prepared nanosilicate-substituted strontium-hydroxyapatite stocks were used in the experiments.

#### 2.5.2. MTT Assay

Human chondrocyte and mouse osteoblast cell lines were seeded at a density of 10,000 cells per 1 cm^2^ in 96-well plates and allowed to attach and grow for 24 h. After obtaining 60–70% of density, the TC28A2 and 7F2 cell lines were washed with sterile PBS (Biowest), then sterile media and various concentrations (50 µg/mL, 100 µg/mL) of tested nanoparticles were added. The MTT (Sigma-Aldrich) assay was performed 24 h after cell treatment with added nanoparticle compounds. The treatment medium was removed and sterile PBS containing 0.5mg/mL MTT (tiazol blue tertazolium) was added. Cells were then incubated for 3 h at 37 °C. After incubation, the medium containing MTT was removed without washing and formed formazan crystals were dissolved in DMSO (Sigma-Aldrich). Absorbance was read at 560 nm with background reference at 670 nm using the plate reader (Varioskan LUX, Thermo Fisher Scientific, Waltham, MA, USA). The whole experiment was repeated three times for each cell line. The viability of used cell lines was estimated via the following formula:

$$\mathrm{Cell}\;\mathrm{viability}=\frac{sample\;absorbance}{control\;absorbance}\times 100$$

### 2.6. Hemolytic Activity

The hemolysis test was performed according to the slightly modified standard protocol [38]. Ram blood (ProAnimali, Wroclaw, Poland) was centrifuged (3000 rpm, 10 min) in order to obtain erythrocyte fraction which was washed with PBS (phosphate-buffered saline) and mixed with fresh PBS (1:1 *v*/*v*). The tested silicate-substituted strontium-hydroxyapatites were suspended in PBS, mixed with erythrocytes (at the final concentration of 50 and 100 µg/mL) and incubated at 37 °C for 24 h. Then, samples were centrifuged to obtain supernatant (5000 RPM, 5 min) and the optical density was measured at 540 nm using a plate reader (Varioskan LUX, Thermo Fisher Scientific, Waltham, MA, USA). The solution of 1% SDS (sodium dodecyl sulfate) and saline were used as a reference and as negative controls, respectively. Statistical analysis was performed using a one-way ANOVA test (*p* < 0.05). The hemolysis percentage was calculated using the formula below:

$$Hemolysis=\frac{sample\;absorbance\;-\;negative\;control\;absorbance}{positive\;control\;absorbance\;-\;negative\;control\;absorbance}\times 100$$

Then, the erythrocytes were smeared on the glass slide and observed under the microscope to evaluate the effect of silicate-substituted strontium-hydroxyapatite on blood cell morphology (OlympusIX83 Fluoview FV 1200, camera CCD Hamatsu C13440, magnification 20×).

### 2.7. Ames Test

The Ames test was chosen to investigate the mutagenic potential of the tested silicate-substituted strontium-hydroxyapatites [39]. For this purpose, two standard bacterial strains were used: *Salmonella* Typhimurium TA98 and *Salmonella* Typhimurium TA100. Minimal Davis medium was, according to the procedure, described elsewhere [40] and supplemented with a 20% solution of glucose. The bacterial suspensions in Luria Broth medium (optical density of 1.5) were diluted 20× in agar solution (0.6%), mixed with 0.5% of NaCl and a 10% *v*/*v* mixture of D-biotin (0.3 mg/mL) and L-histidine (0.5 mg/mL) solutions. The obtained top agars were spread on the surface of solid minimal Davis medium and incubated for 48 h at 37 °C. As a positive control the solutions of 15 µg/mL of sodium azide (for *S*. Typhimurium TA 98) and 100 µg/mL of acriflavine (for *S*. Typhimurium TA 100) were applied. As a negative control the saline solution was used. After incubation the colonies were counted, and the MR (mutagenic ratio) was calculated as follows:

$$MR=\frac{number\;of\;revertants\;formed\;after\;incubation\;with\;tested\;compounds}{number\;of\;revertants\;formed\;spontaneously\;\left(negativecontrol\right)}$$

## 3. Results

### 3.1. Structural and Morphology Analysis

**Figure 1:**
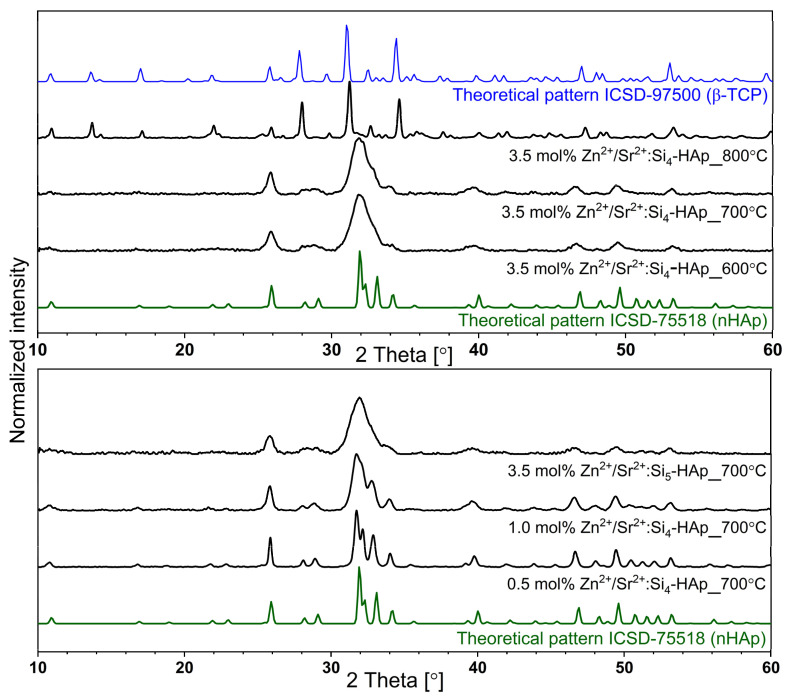
XRD patterns of the silicate-substituted apatite of all investigated samples as a function of the heat-treated temperature as well as the concentration of doping ions.

The measured X-ray diffraction patterns of the silicate-substituted strontium-hydroxyapatite were compared with the reference pattern of strontium-substituted hydroxyapatite from Inorganic Crystal Structure Database (ICSD-75518). The results were analyzed and are presented in Figure 1. The pure hexagonal lattice structure (space group: *P*6_3_*/m*, number: 176) was obtained for all samples. The most intense diffraction peaks are placed at 25.86° (002), 31.82° (211), 32.16° (300), 32.91° (202), 39.73° (310), 46.66° (222) and 49.44° (213). Materials showed stable hydroxyapatite structure up to 700 °C of the heat-treating temperature. Peaks correlating with additional crystal phase appeared at the XRD pattern of the powder heat-treated at 800 °C. Peaks are corresponded to the β-TCP crystal structure. The theoretical pattern of β-TCP (ICSD-97500) is presented in Figure 1. Therefore, powders heat-treated at 700 °C were chosen for biological activity tests.

The X-ray powder diffraction pattern provides information about the crystals’ size and crystalline quality. It was observed that the full width at half maximum (FWHM) of the peaks increased with an increase of Zn^2+^ ion-dopant concentration. Narrow peaks were observed in the XRD pattern of Zn^2+^-doping concentration below Ca_9.8−x−n_Sr_n_Zn_x_(PO4)_6−y_(SiO_4_)_y_(OH)_2_ (where: n = 0.2 [mol%]; x = 0.5–3.5 [mol%]; y = 4–5 [mol%]). An increase of the FWHM is correlated with a decrease of the average crystal size and unit cell parameters.

**Figure 2:**
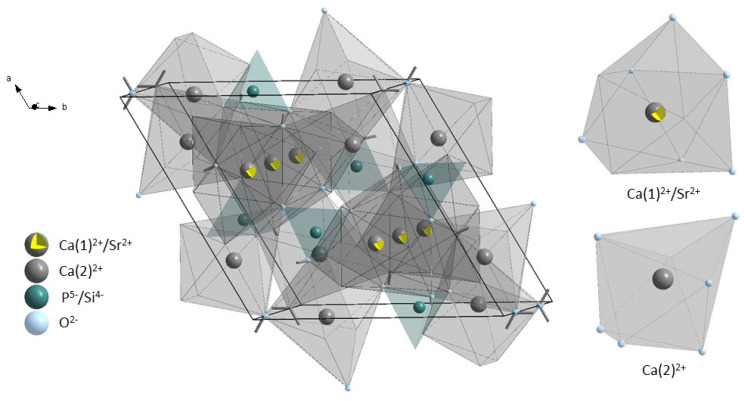
Unit cell representation of silicate-substituted strontium-hydroxyapatite and visualization of polyhedral surrounded calcium ions.

Two different calcium positions with different chemical and structural properties are present in the hydroxyapatite crystal structure. The Ca(1) site is surrounded by nine oxygen atoms from PO_4_^3−^ groups, which formed coordination polyhedron with formula CaO_9_ with C3 symmetry. The Ca(2) site is surrounded by six oxygen atoms from PO_4_^3−^ and one oxygen atom from the hydroxyl group. The Ca(2) site is surrounded by an irregular polyhedron with the formula CaO6OH with Cs symmetry formed by seven oxygen atoms [41,42]. The representation of the unit cell of silicate-substituted strontium-hydroxyapatite and polyhedrons surrounding calcium sites are presented in Figure 2. 

### 3.2. SEM-EDS Analysis

**Figure 3:**
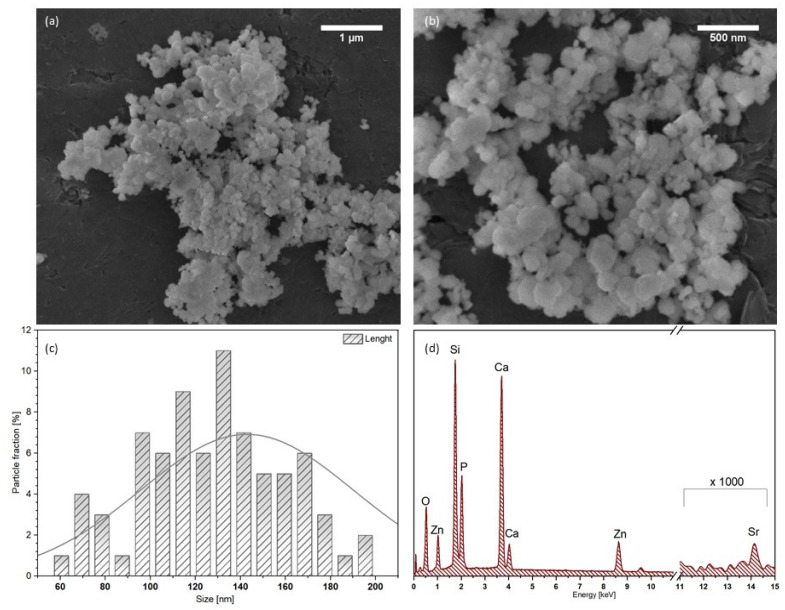
The SEM images (**a**,**b**), particle size distribution (**c**) as well as EDS element analysis (**d**) of 3.5Zn^2+^/Sr^2+^:4Si-HAp heat-treated in 700 °C.

The SEM images and EDS spectra of synthetic silicate-substituted strontium-hydroxyapatite powders were recorded and presented in Figure 3. SEM images demonstrate that obtained powders are of irregular-rounded shaped and particles tend to agglomerate. The self-aggregation process is common in the case of nanocrystals. The forces that are responsible for this feature are associated with intramolecular or intraparticle interactions. The presence of der Waals forces, dipole−dipole force, electrostatic interaction and hydrogen bonds results in the self-aggregation of the particles [43,44]. Figure 3c presents the histogram of the particle size distribution, and the length was measured based on SEM images. The particle size is in a range of 60–200 nm. The concentration of elements in the Ca_9.8−x_Sr_0.2_Zn_x_(PO_4_)_6−y_(SiO_4_)_y_(OH)_2_ (where: x = 0.5–3.5 [mol%] and y = 4–5 [mol%]) was measured using the EDS technique. The content of dopant ions: Sr^2+^ and Zn^2+^ ions, as well as the amount of silicate groups in obtained samples, is in the agreement with the assumptions. The content of dopant ions was calculated using the following equation:

(1) $$X_{n/x}\;\left[\mathrm{mol}\%\right]=\frac{\mathrm{mol}\%\;X\cdot 10}{\mathrm{mol}\%\;({\mathrm{Ca}}^{2+}+{\mathrm{Sr}}^{2+}+{\mathrm{Zn}}^{2+})}$$

The amount of silicate group was calculated using the following equation:

(2) $${\left({\mathrm{SiO}}_{4}\right)}_{y}^{4-}\left[\mathrm{mol}\%\right]=\frac{{\mathrm{mol}\%\mathrm{Si}}^{4+}\cdot 6}{\mathrm{mol}\%\;\left({\mathrm{Si}}^{4+}+P^{5+}\right)}$$

### 3.3. Fourier-Transformed Infrared Spectroscopy

**Figure 4:**
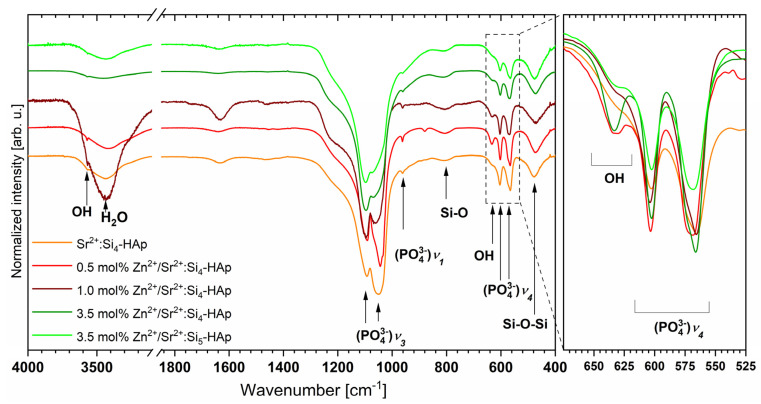
The transmission FT-IR spectra of the silicate-substituted strontium-hydroxyapatite co-doped with Zn^2+^ ion. Spectra recorded for the samples after heat-treating at 700 °C.

The FT-IR transmission spectra were recorded to investigate the chemical bonding of the obtained powders. Figure 4 presents the infrared spectra of the compounds that were heat-treated at 700 °C. The characteristic peaks correspond to the apatites and are in agreement with previous work [45,46,47,48,49].The broad absorption peaks of the bending mode of the silicate group: Si-O-Si were observed at 420–527 cm^−1^. The broad bands corresponding to the O-Si-Omol%] stretching vibes were recorded at 780–842 cm^−1^ for all obtained materials. The difficulty in differentiation of all peaks relating to silicon and phosphate groups in the silicate-substituted strontium-hydroxyapatite could be due to the similarity of the vibes energy of these bonds. The vibration modes related to the silicate group can be overlapped with the phosphate modes, which have been noticed in the following cases:bands corresponding to the bending mode of Si-O-Si and to bending modes of O-P-Owere observed at approximately 470 cm^−1^, and the Si-O symmetric stretching mode and the P-O symmetric stretching mode are detected at approximately 950 cm^−1^ (precisely at 949 cm^−1^ and at 965 cm^−1^, respectively) [49]. The most intense lines related to theν_3_ (PO_4_^3−^) stretching modes are located at 1055 cm^−1^ and at 1095 cm^−1^. Narrow peaks ascribed to the bending modes of ν_4_ (PO_4_^3−^) were detected at 605 cm^−1^ and at 569 cm^−1^ wave numbers as expected, two peaks corresponding to the OH-group vibes were detected in the infrared spectrum of the silicate-substituted strontium-hydroxyapatite samples at 3571 cm^−1^ and at 633 cm^−1^. The presence of those peaks clearly confirms the obtainment of the silicate-substituted hydroxyapatite matrix.

### 3.4. Antibacterial Activity

**Figure 5:**
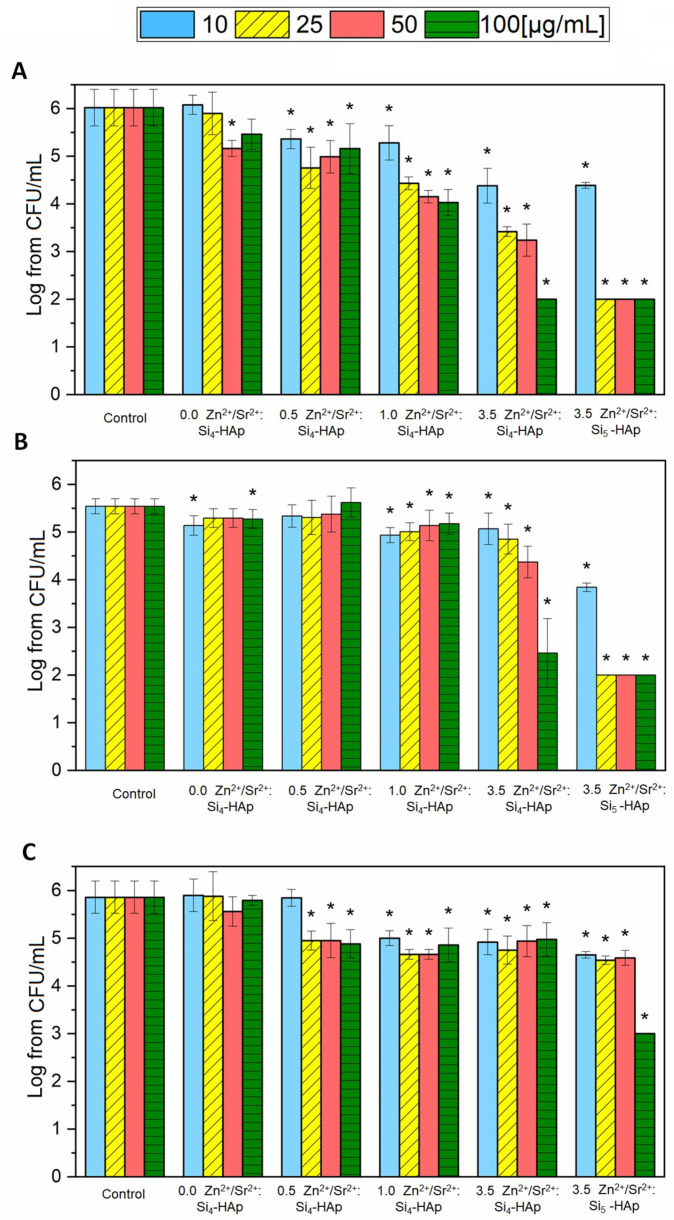
Antibacterial activity of silicate-substituted hydroxyapatite co-doped with Sr^2+^ and Zn^2+^ (0.5, 1.0 or 3.5 mol %) against: (**A**) *K. pneumoniae* ATCC 700603, (**B**) *E. coli* ATCC 35218, (**C**) *P. aeruginosa* ATCC 27853 (mean ± sd, n = 3; *—statistically different from the control (*p* < 0.05).

The antibacterial activity was tested using three Gram-negative strains (*Pseudomonas aeruginosa* ATCC 27853, *Escherichia coli* ATCC 35218 and *Klebsiella pneumoniae* ATCC 700603) that were recommended for susceptibility testing. The antibacterial activity (Figure 5) of the tested series of silicate-substituted strontium-hydroxyapatite was observed mainly against *E. coli* and *K. pneumoniae* strains. *P. aeruginosa* seemed to be the most resistant among all tested bacteria. Silicate-substituted hydroxyapatite doped only with Sr^2+^ did not exhibit antimicrobial activity at the tested concentrations (10–100 µg/mL), but the addition of Zn^2+^ ions, especially 1 and 3.5 mol% dopants, increased its activity. The strongest antibacterial effect was observed for Si-HAp co-doped with Sr^2+^ and 3.5 mol% Zn^2+^. It could be easily seen that the hydroxyapatite modified with five silicone groups was more active than its four-group compound. However, the EDS measurement (see Table 1) revealed that actual Zn^2+^ ion-dopant is higher in the latter, so these two compounds should not actually be compared.

### 3.5. Antibiofilm Activity

**Figure 6:**
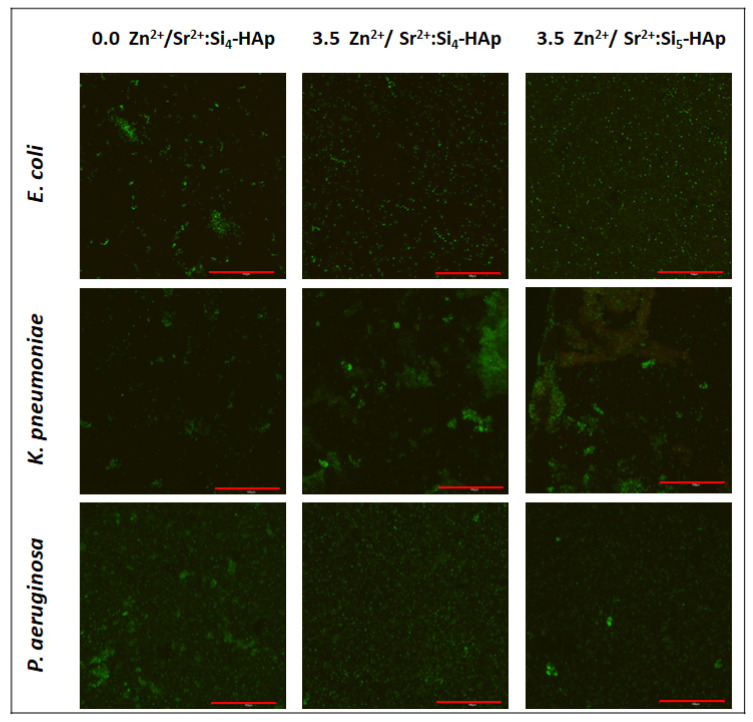
Bacterial biofilm on the surface of pellets (scale bar = 100 µm).

The ability of the biofilm formation is a very important virulence factor of many bacterial species. One of them, *Klebsiella pneumoniae,* produces biofilm for the better colonization of urinary and respiratory tracts. Bacterial cells surrounded by the matrix are less susceptible to antibiotics and disinfectants, as well as harmful environmental conditions, including immune defense mechanisms [50,51]. *P. aeruginosa* and *E. coli* are also known for their high biofilm production [37,52]. Impairments in the bacterial biofilm formation on abiotic surfaces is essential in reducing implant-associated infections cases. The obtained results point to the possibility of the future application of tested materials as coatings of biomedical devices, such as catheters, prosthetics and others [37]. Figure 6 shows the biofilms that formed on the surface of silicate-substituted strontium-hydroxyapatite pellets. Generally, no significant growth reduction was observed for any of tested bacteria. Moreover, the biofilms are mainly composed of living cells (green color). However, it could be seen that the morphology of biofilms is different on the surfaces. In the case of *E. coli* and *P. aeruginosa,* single adhered cells can be observed at Zn^2+^ ion-doped pellets, rather than the more complex biofilm fragments which are present at the Zn^2+^ ion-free sample. *K. pneumoniae*, on the contrary, seems to form more a complex biofilm structure on Zn^2+^ ions-doped materials than on the non-doped one. 

### 3.6. Cytotoxicity Evaluation

**Figure 7:**
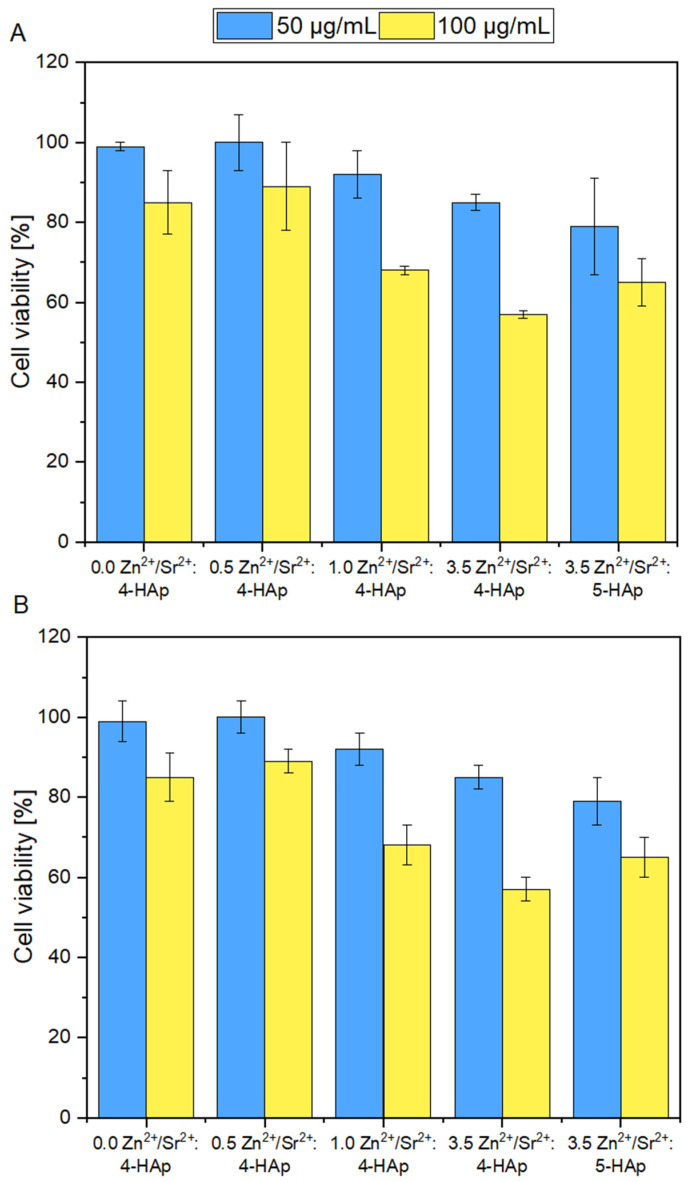
Influence of Zn^2+^ and Sr^2+^ ions co-doped silicate-substituted hydroxyapatite nanoparticles on the viability of (**A**) TC28A2 and (**B**) 7F2 cell lines.

The results clearly indicate that the viability of the TC28A2 and 7F2 cell lines strongly depends on the concentration of the tested compounds. An evident difference in cell viability can be observed between the cells treated with 50 ug/mL and 100 ug/mL. Although the percentage of living TC28A2 and 7F2 cells is maintained at approximately 80% to 100% among all tested compounds when treated with 50 ug/mL, a significant drop of viability is observed when treated with 100 ug/mL (Figure 7); especially, when the concentration of doped Zn^2+^ ions in the nanopowder samples increases. The highest number of viable cells is observed after treatment with 50 µg/mL ofCa_9.3_Sr_0.2_Zn_0.5_(PO_4_)_2_(SiO_4_)_4_(OH)_2_ and Ca_9.3_Sr_0.2_Zn_0.5_(PO_4_)_2_(SiO_4_)_4_(OH)_2_ and still, when cells are treated with 100 ug/mL, the amount of metabolically active cells is maintained highly above 80%. The results presented in Figure 7 also showed that the increased concentration of doped Zn^2+^ ions in silicate-substituted strontium-hydroxyapatite materials causes the reduction of the viability of both TC28A2 and 7F2 cells. The experimental results indicate that neither the concentration of Sr^2+^ ions doped into hydroxyapatite structure of obtained materials, nor the presence of SiO_4_ affect the viability of mouse osteoblast and human chondrocyte cell lines. The results also point out that the more Zn^2+^ ions that are doped into the hydroxyapatite structure and the greater the number of silica groups substituted in hydroxyapatite-based compounds, the more severe the cytotoxic effect among both cell lines is observed. This can be caused by synergic effect of both silica group and zinc ions. However, the substitution of (PO_4_) group with five (SiO_4_) groups instead of four does not seem to have a large effect on cell viability; rather that the Zn^2+^ ions are considered to have bigger impact and disturb cell proliferation (Figure 7). 

### 3.7. Hemolysis

**Figure 8:**
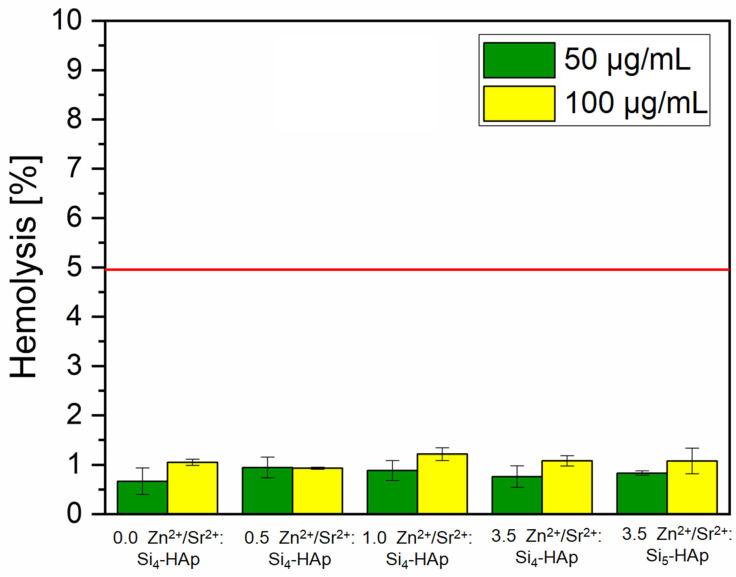
Hemolytic activity of tested silicate-substituted strontium-hydroxyapatite at the concentration of 50 and 100 µg/mL compared with the hemolysis caused by the solution of 1% SDS. The red line indicates approvable hemolysis level (5%). All results were statistically significant (*p* < 0.05).

**Figure 9:**
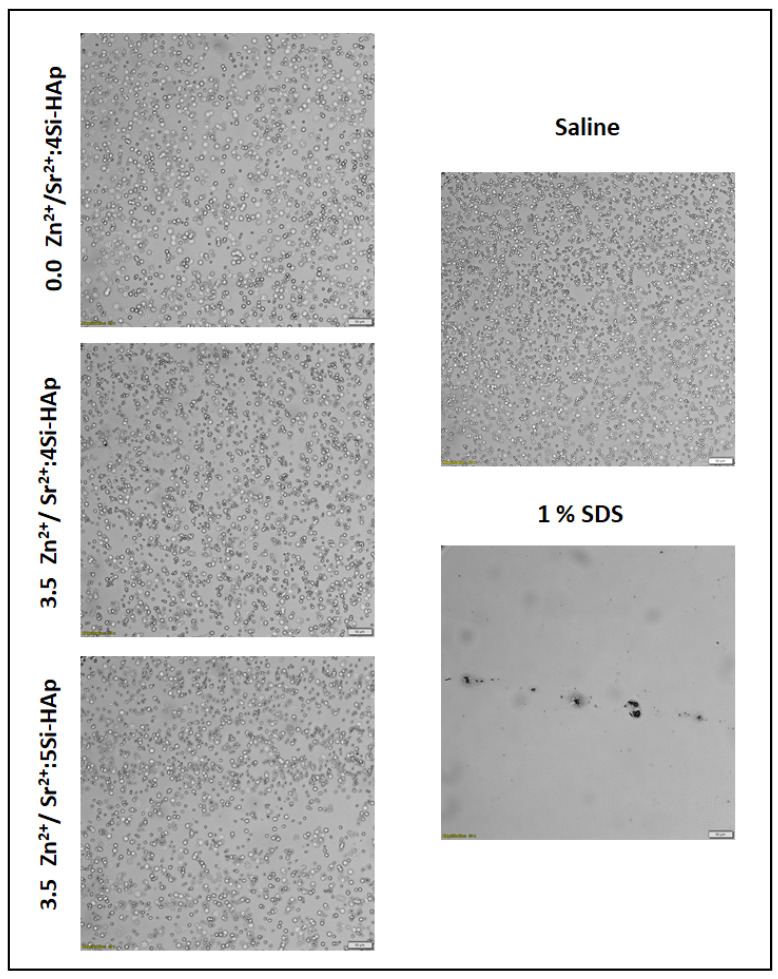
Microscopic photos of erythrocytes after incubation with tested silicate-substituted strontium-hydroxyapatite (scale bar = 50 µm).

Testing the newly synthesized compounds for the hemolytic properties is an essential step in the assessment of their cytotoxicity. This is especially important when the materials are intended to come into direct contact with the human body. The results of hemolysis assay are shown in Figure 8 and in the microscopic images in Figure 9, depicting erythrocyte morphology after incubation in the presence of a tested material. None of the tested compounds caused hemolysis at a level above approvable (5%) [53]. Moreover, the microscopic observations revealed that none of the tested compounds caused visible changes in erythrocyte morphology (Figure 9). The results confirm the non-hemolytic activity of the tested materials.

### 3.8. Ames Test

The Ames test is the well-known method used for the assessment of the mutagenic properties of different substances. In this research, none of the tested silicate-substituted strontium-hydroxyapatites showed a mutagenic ratio above the acceptable level of 1.7 (Table 2), hence it is clearly visible that these materials, at the tested concentration, do not have mutagenic properties.

## 4. Discussion

Since synthetic hydroxyapatites are commonly used in many clinical aspects, modification of their structure for the enhancement of its properties is an obvious approach [54]. The doping of HAp with metal ions might not only increase tissue regeneration but also provide antimicrobial properties; thus studies regarding various potential dopants (single or multiple) are indeed needed. The implantation procedures bear a risk of an infection, mainly of bacterial origin, with *P. aeruginosa* and *E. coli* being one of the most common nosocomial pathogens among Gram-negative bacteria [37]; however, such infections are also noted for *K. pneumoniae* [55,56].

The antibacterial activity of hydroxyapatites doped with Zn^2+^ ion was previously thoroughly investigated and obtained data suggest the highest bacterial growth reduction at the Zn^2+^ ion concentration of 1–2 mol% [5]. Moreover, activity against *E. coli* was confirmed for colloidal solutions of Ca_10−x_Zn_x_(PO_4_)_6_(OH)_2_ (x = 0.0, 0.07 or 0.2) at concentrations from 1.95 µg/mL up to 1 mg/mL [57]. In our previous work, the Zn^2+^-doped hydroxyapatite (5 mol%) was tested against *P. aeruginosa* and *E. coli* and no activity was spotted. Hence, in the present work, we investigated antibacterial activity of HAp co-doped with Zn^2+^ against three Gram-negative strains: *P. aeruginosa*, *E. coli* and *K. pneumoniae* and we decided to lower the Zn^2+^ content to 3.5 mol%. Moreover, the Sr^2+^ was added to enhance water solubility [58]. Even though HAp doped with strontium alone did not show any antibacterial effects, co-doping with Zn^2+^ ions significantly reduced bacterial growth, especially in the cases of *E. coli* and *K. pneumoniae*. Although nanoparticles of ZnO have well-documented activity against *P. aeruginosa*, this species appeared to exhibit a higher tolerance to Zn^2+^ ions released from HAp, which was also shown by Karetsi et al., 2019 [59]. 

In the hospital environment, nosocomial pathogens may adhere to surgical devices or implanted materials, and once adhered, they may develop into the biofilm, a three-dimensional bacterial community with an increased resistance to drugs [60,61]. Thus, the exhibition of antibiofilm properties of grafted materials would be an additional advantage. Apart from growth inhibition caused by the local release of metal ions, the material structure might be modified to reduce the adhesion of hazardous bacteria. However, our research showed that there was no significant effect of HAp doped with Sr^2+^ and Zn^2+^ on bacterial biofilm reduction or survival. This results are to be expected, since the surface of HAp is porous, which might facilitate interaction with bacterial cells [62], a phenomenon often observed in the case of dental plaque development [63]. Although some changes in the biofilm architecture could be seen after co-doping with Zn^2+^ ions, it should be noted that the most probable reason for such slight alterations is the difference between particular pellets’ surfaces. In our previous research, it was proved that generally the surface of hydroxyapatite pellet is smooth, which prevents bacteria adherence but when the surface is “rough” (more porous), the bacterial (and cellular) attachment is greater [19]. Thus, it can be assumed that the differences observed between particular materials in the present research may be the result of differences in the pellets’ surfaces which cannot be avoided using the hand press in the preparation process.

However, although the anti-biofilm properties of the newly synthesized materials are highly desirable, one of the most important features should be also the lack of cytotoxicity. Hydroxyapatite nanoparticles are known to be an inseparable part of the vertebrate skeletal system and bone structure. Hydroxyapatites along with collagen fibers create natural and solid bone framework and, therefore, many studies have proved the non-cytotoxic character of synthetic HAp nanoparticles. Prior to medical application, all materials and substances must be conducted to toxicity testing (e.g., cytotoxicity, mutagenic potential or effects on blood cells) to verify their safety. The cytotoxicity studies of Sr^2+^/Zn^2+^ doped Si-HAp showed no toxic effects at the lower concentration. However, cell viability decreased when Zn^2+^ ions amount in the Si-HAp structure was elevated. These results agree with previously published data. Qiu et al. (2006) showed that strontium-doped calcium polyphosphate scaffolds (even with 100% of strontium substitution for calcium) were not toxic against the proliferation of osteoblastic cell lines [64]. Similarly, when Ni et al. (2011) doped hydroxyapatite with Sr^2+^, it not only caused no loss in the cell viability but also increased the mineralization and differentiation processes [65]. The Zn^2+^ ion, on the other hand, was proved to have a significant influence on cell viability, since rather low concentrations of Zn^2+^ are able to reduce cell viability, adhesion and increase reactive oxygen species (ROS) production [66]. The present study shows that only a low amount of Zn^2+^ ion-dopant might be introduced to the HAp structure to consider this material as safe, and these results are also supported by other published data [67,68]. Introducing silicate into hydroxyapatite might also influence its toxicity. Numerous studies indicate that the toxicity of silica nanoparticles strongly depends on their size and concentration [69,70,71]. Our results may indicate that an increase in the number of silica groups from four to five causes some reduction of cell viability; however, a decreased number of living cells was caused rather by Zn^2+^ ions than in (SiO_4_) groups substituted by (PO_4_). Hemolysis assay is a crucial step of safety investigation of newly synthesized materials. Generally, apatite-based materials are considered biocompatible and are not expected to exhibit any significant hemolytic activity [72,73], but the confirmation is necessary, especially for materials co-doped with metal ions. Sr^2+^/Zn^2+^-doped HAp did not cause erythrocyte disruption and hemoglobin release. No mutagenic potential showed in the Ames test which also points at the safety of the tested materials, corresponding with previously published data [74].

## 5. Conclusions

This study is the first successful obtainment of the silicate-substituted hydroxyapatite co-doped with Sr^2+^ and Zn^2+^ ions by the hydrothermal method assisted with the microwave technique. The pure hexagonal structure has been confirmed by XRD analysis. The nominal Si content incorporated in the PO_4_^3−^ sites of the HAp lattice cell is in agreement with the theoretical value. The FT-IT spectra present characteristic absorption lies corresponds with the chemical bands of HAp molecules. Silicate-substituted strontium-apatites co-doped with Zn^2+^ ion exhibit dose-dependent antibacterial activity against *Klebsiella pneumoniae* and *Escherichia coli*. No inhibition of the biofilm formation was observed. However, biofilm morphology is different for the Zn^2+^ ion-doped and Zn^2+^ ion-free pellets. Tested materials do not possess hemolytic and mutagenic properties. Furthermore, they cause dose-dependent cytotoxicity towards chondrocytes and osteoblasts. The results obtained for the antimicrobial and cytotoxic studies correlate and prove that the biological activity of Sr^2+^/Zn^2+^-doped Si-HAp increased with the increase of Zn^2+^ ions content as well as with the number of silica groups. However, the number of silica groups only has a visible impact in antibacterial tests.

## Figures and Tables

**Table 1:** Results of principal components measured using the EDS technique.

Sample	Theoretical Chemical Formula	EDS Technique		
		n Sr^2+^ [mol%]	x Zn^2+^ [mol%]	y (SiO_4_)^4−^ [mol%]
0.0 mol% Zn^2+^/Sr^2+^:Si_4_-HAp	Ca_9.8_Sr_0.2_(PO_4_)_2_(SiO_4_)_4_(OH)_2_	0.19	0.00	3.40
0.5 mol%Zn^2+^/Sr^2+^:Si_4_-HAp	Ca_9.3_Sr_0.2_Zn_0.5_ (PO_4_)_2_(SiO_4_)_4_(OH)_2_	0.15	0.33	3.78
1.0 mol%Zn^2+^/Sr^2+^:Si_4_-HAp	Ca_8.8_Sr_0.2_Zn_1_ (PO_4_)_2_(SiO_4_)_4_(OH)_2_	0.13	1.12	3.64
3.5 mol%Zn^2+^/Sr^2+^:Si_4_-HAp	Ca_6.3_Sr_0.2_Zn_3.5_(PO_4_)_2_(SiO_4_)_4_(OH)_2_	0.17	2.49	3.79
3.5 mol%Zn^2+^/Sr^2+^:Si_5_-HAp	Ca_6.3_Sr_0.2_Zn_3.5_(PO_4_)(SiO_4_)_5_(OH)_2_	0.15	3.44	4.23

**Table 2:** Mutagenic ratio of tested silicate-substituted strontium-hydroxyapatites calculated for *S.* Typhimurium TA98 and TA100 strains.

Compound	Concentration [μg/mL]	*S*. Typhimurium TA98	*S*. Typhimurium TA100
		Mutagenic Ratio (MR)	Mutagenic Ratio (MR)
Negative control		1.00	1.00
Positive control		13.20	9.14
0.0 mol% Zn^2+^/Sr^2+^:Si_4_-HAp	50	0.88	1.31
	100	0.68	1.42
0.5 mol% Zn^2+^/ Sr^2+^:Si_4_-HAp	50	0.90	1.00
	100	0.78	1.04
1.0 mol% Zn^2+^/ Sr^2+^:Si_4_-HAp	50	0.78	1.35
	100	0.80	0.98
3.5 mol% Zn^2+^/ Sr^2+^:Si_4_-HAp	50	0.90	1.02
	100	0.78	1.25
3.5 mol% Zn^2+^/ Sr^2+^:Si_5_-HAp	50	0.82	1.09
	100	0.83	1.40

## Data Availability

Not applicable.
